# Rhenium Radioisotopes for Medicine, a Focus on Production and Applications

**DOI:** 10.3390/molecules27165283

**Published:** 2022-08-18

**Authors:** Licia Uccelli, Petra Martini, Luca Urso, Teresa Ghirardi, Lorenza Marvelli, Corrado Cittanti, Aldo Carnevale, Melchiore Giganti, Mirco Bartolomei, Alessandra Boschi

**Affiliations:** 1 Department of Translational Medicine, University of Ferrara, 44121 Ferrara, Italy; 2 Nuclear Medicine Unit, University Hospital, 44124 Ferrara, Italy; 3 Department of Environmental and Prevention Sciences, University of Ferrara, 44121 Ferrara, Italy; 4 Department of Chemical, Pharmaceutical and Agricultural Sciences, University of Ferrara, 44121 Ferrara, Italy; 5 Radiology Unit, University Hospital, 44124 Ferrara, Italy

**Keywords:** rhenium-186, rhenium-188, radiotherapy

## Abstract

In recent decades, the use of alpha; pure beta; or beta/gamma emitters in oncology, endocrinology, and interventional cardiology rheumatology, has proved to be an important alternative to the most common therapeutic regimens. Among radionuclides used for therapy in nuclear medicine, two rhenium radioisotopes are of particular relevance: rhenium-186 and rhenium-188. The first is routinely produced in nuclear reactors by direct neutron activation of rhenium-186 via ^185^Re(n,γ)^186^Re nuclear reaction. Rhenium-188 is produced by the decay of the parent tungsten-188. Separation of rhenium-188 is mainly performed using a chromatographic ^188^W/^188^Re generator in which tungsten-188 is adsorbed on the alumina column, similar to the ^99^Mo/^99m^Tc generator system, and the radionuclide eluted in saline solution. The application of rhenium-186 and rhenium-188 depends on their specific activity. Rhenium-186 is produced in low specific activity and is mainly used for labeling particles or diphosphonates for bone pain palliation. Whereas, rhenium-188 of high specific activity can be used for labeling peptides or bioactive molecules. One of the advantages of rhenium is its chemical similarity with technetium. So, diagnostic technetium analogs labeled with radiorhenium can be developed for therapeutic applications. Clinical trials promoting the use of ^186/188^Re-radiopharmaceuticals is, in particular, are discussed.

## 1. Introduction

Nuclear medicine is a specific branch of medicine that uses radionuclides to perform diagnostic or therapeutic procedures. Radionuclides applied for diagnosis are those emitting β^+^ particles in PET (Positron Emission Tomography) and γ rays in SPECT (Single Photon Emission Tomography). On the contrary, in the field of cancer therapy, radionuclides that emit energetic α, β^−^ and auger electron particles are used. Since these particles are fully deposited within a small range of tissue (in the range of nm-mm depending on particle mass and energy), they are able to destroy cells responsible for pathologies thanks to the biological effects of the emitted radiation ensuring maximum treatment efficiency limited to the malignant area [1,2,3]. Nowadays, radionuclide therapy has caught great interest in cancer treatment for several reasons: (1) the radioactivity is delivered selectively on the target tumor saving the surrounding normal tissue; (2) minimal radiopharmaceutical administration allows for fast and minimally invasive treatment; (3) all organs or tissues are efficiently achievable from the body inside rather than whole-body irradiation using external beam radiotherapy.

The most commonly used therapeutic radionuclides are iodine-131 (^131^I) to treat thyroid-related diseases; strontium-89 (^89^Sr) and samarium-153 (^153^Sm) labeled radiopharmaceuticals for the treatment of bone metastasis; and rhenium-186,188 (^186^Re, ^188^Re), yttrium-90 (^90^Y), and lutetium-177 (^177^Lu) for Peptide Receptor Radionuclide Therapy (PRRT) and for the treatment of a variety of malignancies, etc. [4]. Among these radionuclides, rhenium radioisotopes are very attractive for several reasons. First of all, they have both particle emissions with properties suitable to perform targeted therapy and photon emissions, which can be used for diagnostic investigation by single photon emission tomography (SPECT). In addition to the nuclear properties, useful for the development of theranostic radiopharmaceuticals, rhenium is part of the same chemical family as technetium; consequently, a large portion of the chemistry developed for technetium can be applied also to rhenium.

This review aims to provide an overview on rhenium radioisotopes for medical application. Most common production methods and the chemical–physical characteristics making these radioisotopes very attractive for tumor therapy are overviewed, and clinical trials promoting the use of ^186/188^Re-radiopharmaceuticals are particularly discussed.

## 2. Rhenium-186 Nuclear Properties and Production

**Figure 1:**
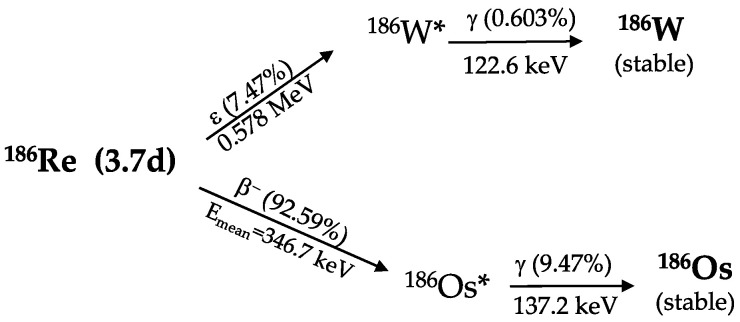
^186^Re simplified decay scheme.

^186^Re (t_1/2_ = 3.7183 d) decays to ^186^W and ^186^Os by electron capture (7.47%) and β^−^ particle emission (92.53%), respectively (Figure 1) [5]. ^186^Re with its low energy beta emission and the maximum tissue penetration of 4.5 mm is a promising candidate for therapy of tumors from millimeter to centimeter dimensions.

It also emits a low-abundance (9.47%) 137 keV γ-ray, suitable for in-vivo imaging of radiolabeled biomolecules and dosimetry studies via SPECT [6]. In Table 1, detailed nuclear decay data are reported [5,7].

^186^Re is routinely produced at specific activities of 3–4 Ci/mg in nuclear reactors by direct neutron activation of metallic ^185^Re via the ^185^Re(n,γ)^186g^Re nuclear reaction [8], and is successfully used for the ^186^Re-HEDP preparation where a low specific activity radiopharmaceutical is required to treat pain from bone metastases.

Despite its half-life making it potentially suitable also for employment in targeting pharmacokinetically slow agents like antibodies, its disadvantage is precisely its low specificity activity due to its production route via neutron irradiation of ^185^Re. Due to its favorable decay mode and its potentiality in therapeutic treatment, alternative methods for producing high specific activity ^186^Re are being explored.

There are several possible ways to produce ^186^Re with an accelerator, via the ^186^W(p, n)^186^Re or ^186^W(d, 2n)^186^Re reactions, irradiating an enriched target in ^186^W with proton or deuterons respectively, or via the ^189^Os(p,α)^186^Re or ^192^Os(p,α3n)^186^Re reactions, irradiating isotopically enriched targets of osmium [10]. All these methods have the advantage to use a target material different from rhenium, which can be chemically separated after the irradiation.

**Figure 2:**
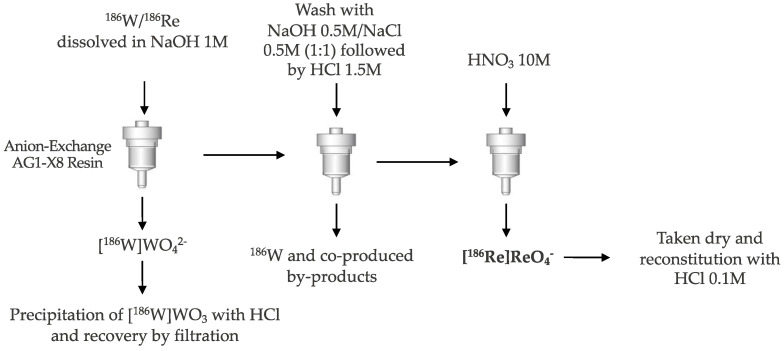
^186^Re processing scheme.

The ^186^W(p, n)^186^Re reaction using a highly enriched target seems to be the method of choice for the production of no-carrier-added ^186^Re taking into account the radionuclidic purity achievable with 18 MeV recommended proton energy [11]. It was found that the optimum production of ^186^Re can be achieved by irradiating natural W target in the energy range 7–14 MeV; whereas, at 15.9 MeV the integral yield obtained was 1.25 MBq·μA^−1^·h^−1^, which could be increased up to 4.32 MBq·μA^−1^·h^−1^ using 99% enriched ^186^W-target [11]. The reaction with deuterons offers, however, the advantage of higher reaction cross sections and lower levels of co-produced isotopic impurities, achieving four times higher yield of ^186^Re at deuteron energy of 20 MeV compared to the (p,n) reaction at 18 MeV, and an amount of ^185^Re formed about 55% of the value associated with the (p,n) process. Recent studies have shown the difficulty of ^186^Re large-scale production using the ^186^W(d,2n)^186^Re reaction on W metal and WO_3_ targets [12]. Targets prepared from WO_3_ were found to be unstable under the irradiation conditions of 10 min total integrated current at 10 µA with a nominal extracted deuteron energy of 17 MeV, while W metal targets encased in graphite withstood up to 10 μA beam currents for 10 min without degradation. The scale-up ^186^Re production via the ^186^W(d,2n)^186^Re reaction was subsequently obtained using irradiated natural abundance and enriched thick ^186^W powder targets encased in graphite layers. The irradiations were conducted at higher beam currents (20 and 27 μA) and with an irradiation time of up to 2 h [13]. In this case, it is important to develop a target recycling strategy of the costly enriched ^186^W targets. This can be conducted through a one-step anion exchange-based chemical recovery method as schematized in Figure 2 [13].

The separation of radiorhenium from oxide or metallic matrices can be also carried out using methods other than anion-exchange chromatography [6] such as liquid-liquid extraction with methyl ethyl ketone (MEK) [14] from target alkaline NaOH solution and thermo-chromatography [15,16].

## 3. Rhenium-188 Nuclear Properties and Production

**Figure 3:**
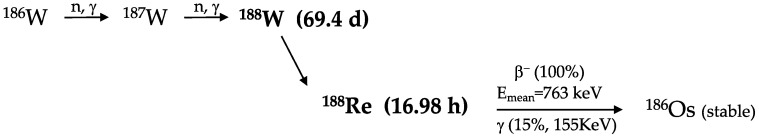
^188^Re simplified production and decay scheme.

Rhenium-188 (^188^Re, t_1/2_ = 17.005 h) is an excellent candidate for the development of radiopharmaceuticals for therapy since it decays 100% emitting β particles with a maximum tissue penetration of 11 mm, suitable for treating large size tumor, and γ photons (E_γ_ = 155 KeV) detectable for scintigraphy and used to test the effectiveness of treatment and absorbed radiation dose. Unlike ^186^Re, its relatively short half-life would seem to limit ^188^Re use to agents with fast target absorption and non-target tissue clearance, while the former can also be used in targeting agents with longer biological half-lives, such as antibodies. However, the possibility of producing ^188^Re at higher specific activities makes it particularly attractive even for radioimmunotherapy applications [17]. ^188^Re is produced in high specific activity by the decay of parent wolfram-188 (^188^W, t_1/2_ = 69.4 d), which is produced through a double neutron capture reaction on ^186^W, according to the diagram in Figure 3. In Table 2, detailed nuclear decay data are reported [7,9].

The peculiar characteristic of being produced by the decay of a parent nuclide allowed to develop the ^188^W/^188^Re generator system similarly to the technetium-99m, providing a long-term source for non-carrier added (nca) ^188^Re to the Department of Nuclear Medicine [18,19]. Unfortunately, unlike the technetium-99m generator, the limited availability and high cost of GMP pharmaceuticals grade-generators have greatly limited the development of therapeutic agents based on ^188^Re.

**Figure 4:**
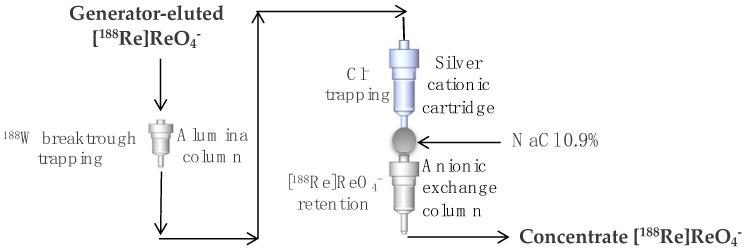
^188^Re post-elution concentration system.

The ^188^W/^188^Re generator system is based on an alumina column, where the long-lived parent ^188^W is adsorbed while radiorhenium is eluted with sterile saline solution in the form of sodium perrhenate ([^188^Re]ReO_4_Na). Thanks to its rapid in-growth, about 60% in 24 h, ^188^Re can be eluted daily and used for optimal clinical use of ^188^Re-radiopharmaceuticals. Unlike the ^99^Mo/^99m^Tc generator, the low specific activity of the reactor-produced ^188^W (4–5 Ci/g) [20] involves the use of higher amounts of alumina and consequently higher elution volumes not always suitable for therapeutic treatments. Post-elution concentration step, based on a two-column tandem flow-through system (Figure 4), can be used to provide very high radioactive concentration solutions of ^188^Re for radiolabeling (>0.7 Ci/mL saline from a 1 Ci generator) [21]. The concentration system is generally based on the ^188^Re-perrhenate selective retention on a tiny anion exchange column and subsequent recovery in a small volume of normal saline used as eluent. This is possible only when the generator-eluted perrhenate is free of any anionic species such as the chloride anions of the saline solution. For this reason, before being adsorbed on the anionic exchange column, the ^188^Re-perrhenate solution is passed first through a small alumina column and then through a silver cationic cartridge for trapping the chloride anions on which they precipitate like silver chloride (Figure 4).

Alternative methods for post-elution concentration of ^188^Re-perrhenate have also been reported. Mushtaq et al. [22] developed a simple method based on a single diethyl amino ethyl cellulose (DEAE) anion exchanger column. An amount of 20–25 mL of the generator eluted ^188^Re-perrhenate solution in acidic ammonium acetate is trapped in the small anion exchange column of DEAE cellulose and subsequently recovered in 4 mL of NaCl 0.9% with a radiochemical yield of 98% and ^188^W breakthrough less than 10^−3^% [22].

Chakravarty et al. [23] identified an electrochemical approach for the concentration of the generator-produced ^188^Re for radiopharmaceutical applications. The concentration of ^188^Re involves electrodeposition of ^188^Re on a platinum cathode, according to the following reaction [24]:

ReO_4_^−^ + 8H^+^ + 7e^−^ → Re + 4H_2_O   E = +0.362 V

They found that the electrochemical method is a simple and effective way for the concentration of ^188^Re from a dilute solution, leaving ^188^W in the solution under the developed electrochemical conditions as ^188^W cannot be deposited in an aqueous medium. Even a very low radioactive concentration of ^188^Re in the saline medium could easily be concentrated by this method. The main advantage of this method is the simplicity of a single electrolysis step, which may be an alternative to chromatographic systems to provide clinical grade ^188^Re.

## 4. Chemistry of Rhenium Radiopharmaceuticals

Rhenium belongs to group 7 of the periodic table of elements together with manganese and technetium. However, the similarity between them is limited to the formation of a number of simple and identical stoichiometry complexes such as metal (VII) ions (MnO_4_^−^, TcO_4_^−^ and ReO_4_^−^) and oxides (MnO_2_, TcO_2,_ and ReO_2_). The chemistry of rhenium is very rich and closer to that of technetium, respecting the tendency of the elements of the second and third transition series to have more similar chemical–physical characteristics than those of the first series: this behavior is linked to the phenomenon of “lanthanide contraction”. This causes the metals of the third transition series to be smaller than expected, the atomic rays of these elements are very similar to that of their respective congeners belonging to the second period. As an example, it can take the measurements of the crystals radii of tetra-coordinated complexes of Mn(VII), Tc(VII), and Re(VII): 0.39, 0.51, and 0.52 Å, respectively [25]. Likewise, the length of the M-Cl bond in complexes [Tc(IV)Cl_6_]^2−^ and [Re(IV)Cl_6_]^2−^ is 2.35 ± 0.01 Å and the radius of Re(0) and Tc(0) in free metals is identical [25]; therefore, it is possible to predict that analogs complexes of rhenium and technetium may present the same shape, size, charge, dipolar moment, ion mobility, and lipophilia, and thus have the same physical characteristics. A major difference between Re and Tc regards the standard reduction potentials as described in Table 3.

In nuclear medical applications, this becomes extremely important. In fact, in terms of physical properties dependent on shape, size, and charge, analogous rhenium and technetium complexes are identical and cannot be discriminated by biological environments, fluids, or receptor systems. Therefore, ^186/188^Re radiopharmaceuticals could be designed on the basis of the characteristics expressed by some well-developed ^99m^Tc tracers that selectively accumulate in abnormal or tumor tissues, and are used in nuclear medicine as diagnostic agents. In particular, it is extremely important to produce ^186/188^Re complexes that maintain a high specificity against cancer or injury. Thus, the radiation dose would be selectively transferred to the area of interest without damaging normal tissue.

However, it should be borne in mind that analogous ^99m^Tc and ^186^Re complexes do not always show the same biodistribution in vivo. In fact, whereas ^99m^Tc-radiopharmaceuticals are prepared in the concentration order of 10^−8^–10^−6^ M, at “no carrier added” level, ^186^Re-radiopharmaceuticals contain a large amount of carrier and the concentration of rhenium in the final radiopharmaceutical preparation is around 10^−3^ M. The presence of carrier can significantly change the radiopharmaceutical preparation and affect the chemical species that are formed with consequences on its biodistribution. The same bone pain palliation ^188^Re and ^186^Re-HEDP radiopharmaceuticals do not show the same biodistribution if prepared starting from “no carrier free” ^186^Re and from “no carrier added” ^188^Re. The presence of a carrier in ^186^Re leads to the formation of a mixture of polymeric species responsible for the peculiar localization in bone tissue. These species are not formed in high yield if the ^188^Re-HEDP preparation occurs in the absence of a carrier, and in order to have good bone fixation, cold rhenium needs to be added [26]. Likewise, when comparing the biodistributions of similar agents ^99m^Tc and ^186^Re, the “carrier” effect needs to be taken into account by suitably designed experiments.

Rhenium radiopharmaceuticals preparation is very similar to that of analogs ^99m^Tc radiopharmaceuticals. As with ^99m^Tc-radiopharmaceuticals, to prepare the analogs rhenium compounds, the starting reagent is the tetra oxygenated ion ReO_4_^−^, perrhenate is reacted in the presence of a reducing agent, and an appropriate ligand is used to stabilize the reduced oxidate state in the final complex. However, it is important to keep in mind that, according to standard reduction potentials shown in Table 3, rhenium complexes are harder to reduce than those of technetium and tend to oxidize more easily to perrhenate, being less stable than technetium in reduced oxidation states. For this reason, in order to obtain the final radiocomplex with an acceptable yield for clinical application (>95%), the preparation conditions of rhenium compounds should be generally more drastic.

To solve this problem, a synthesis strategy that involves the use of sodium oxalate or oxalate buffer at pH = 3 in the ^188^Re radiopharmaceuticals preparation was developed [19,27].

High yield production of [^188^Re][ReO(DMSA)_2_] and [^188^Re][ReN(DEDC)_2_] and ^188^Re-peptides conjugates was performed in physiological solution by using oxalate ion, not necessary in the preparation of the analogues ^99m^Tc compounds [28,29,30,31].

The possibility of preparing different rhenium radiopharmaceuticals comes from its very rich chemistry. All oxidation states ranging from +7 (d^0^) to −1 (d^8^) have been found in different rhenium compounds with coordination numbers up to nine. This wide range results from the fact that, as we proceed along the transition series, the number of electrons “d” increases and, in the central zone of the series, these orbitals are not completely bound in an inert electronic structure, but remain totally available for bonding. The high availability of these electrons makes possible not only the formation of complexes in which the metal is in high oxidation states but also the retrodonation of electrons from the metal to the ligand, with consequent stabilization of low oxidation states [32].

**Figure 5:**
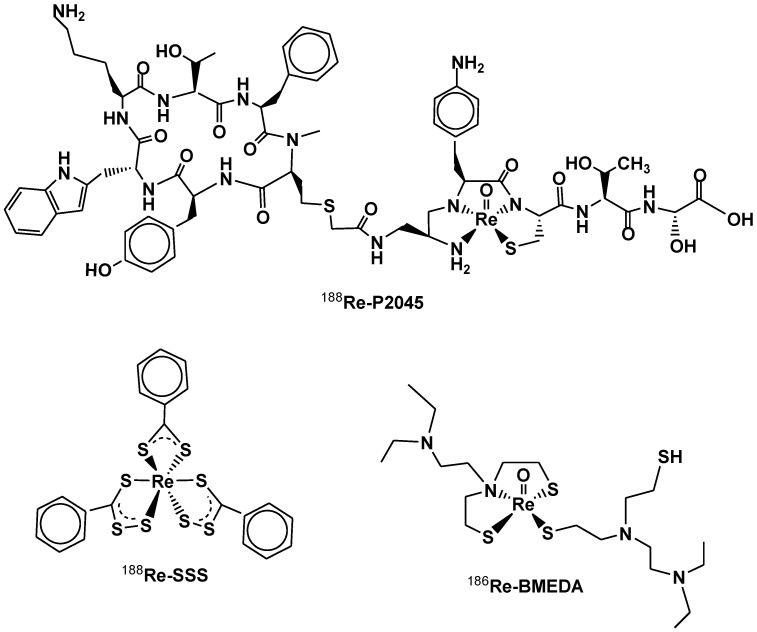
Chemical structure of ^188^Re-P2045, ^188^Re-SSS, and ^186^Re-BMEDA radiopharmaceuticals.

In the +7-oxidation state, rhenium forms a very stable tetrahedral ion ReO_4_^−^, the conjugate base of perrhenic acid (HReO_4_). The ion ReO_4_^−^ represents the starting material for all preparations using this metal, both for nuclear medicine application and in macroscopic synthesis. Oxidation states V and VI are easily dismutated, however, Re(V) is stabilized by numerous ligands, such as N3S and S2N-type ligand that coordinate the “core” (ReO)^3+^, and are successfully used to prepare the ^188^Re-P2045 and ^186^Re-BMEDA/^186^Re-BMEDA [33] radiopharmaceuticals (Figure 5), the applications of which are described in the next section. The tendency to form multiple bonds with oxygen and nitrogen is strong in the range of oxidation states between VII and V, decreasing for IV [32]. The Re-Re bond appears in the complexes of Re(IV) but becomes important to the Re(III). However, mononuclear ^188^Re(III)-SSS radiopharmaceuticals (Figure 5) have been developed for labeling Lipiodol to treat hepato-cellular carcinomas [34].

## 5. 188/186 Re-Radiopharmaceuticals Clinical Application

Although rhenium was discovered by German researchers in 1925, it took decades and multidisciplinary research to arrive at the first ^188^Re-radiopharmaceutical in humans administration [35]. Although ^188^Re, as well as ^186^Re, have ideal physical characteristics for use in nuclear medicine therapy [36,37], the first clinical studies in humans have concerned possible diagnostic applications [38,39,40]. A lot of literature in the past decades has been dedicated to research aimed at identifying ^188^Re or ^186^Re-radiopharmaceuticals to be used in nuclear medicine clinical practice [41], but, at this time, no monographs dedicated to ^188^Re or ^186^Re-radiopharmaceuticals have been published in the European Pharmacopoeia 10th edition yet, and only a few radio-rhenium compounds or devices have been studied in humans. The translational nature of the research is certainly very conditioned by the high production costs of rhenium radionuclides intended for nuclear medical therapeutic use. At the same time, intellectual properties, economic considerations, pharmaceutical companies’ strategies, etc., also weigh on the radiopharmaceutical success that has been shown to be effective in the fight against cancer in preclinical studies.

In this section, we are reporting information relating to recent clinical trials, both completed or still active, and ^188/186^Re-currently used radiopharmaceuticals in clinical routine practice. For this purpose, we consulted with the platform (https://clinicaltrials.gov, last accessed 11 May 2022) [42], nowadays considered the most complete database for clinical trials.

Until now, a total of 17 clinical studies have been found in the US National Library of Medicine with the keyword “Rhenium” (https://clinicaltrials.gov, last accessed 11 May 2022) [42], but only 14 experimental projects target the study of compounds labeled with ^86^Re or ^188^Re (Table 4). In this selection, clinical trials concern n. 9 radiopharmaceuticals (Phase I/II/III/IV): ^188^Re-resin (Rhenium-SCT^®^); ^188^Re-HEDP; ^188^Re-P2045; ^186^Re-NanoLiposomes; ^186^Re-Sulfide; ^188^Re-SSS Lipiodol; ^188^Re labeled anti-HER2 sdAb; ^188^Re-Complex Coupled to an Imidazolic Ligand and Associated With a Dendrime; and ^186^Re-labelled humanized monoclonal antibody BIWA 4.

The radiopharmaceuticals used in the most recent trials are analyzed in more detail below.

### 5.1. “Maximum Tolerated Dose, Safety, and Efficacy of Rhenium Nanoliposomes in Recurrent Glioma” and “Intraventricular Administration of ^186^Re-NanoLiposome for Leptomeningeal Metastases”

Glioblastoma is the most common and most aggressive among the primary malignant brain tumors in adults. To date, external beam radiation is the main therapy for the treatment of primary brain tumors, but it is limited by the tolerance of the surrounding normal brain tissue. In preclinical studies, the Nanoliposomal BMEDA-chelated-^186^Rhenium (^186^Re-NanoLiposome) showed excellent retention in the tumor, allowing the release of beta-emitting radiation of high specific activity [44,45]. A first volume and dose escalation study (Phase I and Phase II; NCT01906385, Recruiting) aims to determine the safety, tolerability, and distribution of ^186^Re-NanoLiposome (NL) administered through a convection-enhanced delivery catheter, in patients with recurrent or progressive malignant glioma after standard surgical treatment, radiotherapy, and/or chemotherapy [46,47]. The immediate goal of this trial is to use high-dose radiation to slow and potentially prevent subsequent recurrent disease, with the ultimate goal of significantly extending patient survival without further surgical resections. A second study (Phase I; NCT05034497, Recruiting) is similar to the previous one but deals with the treatment of leptomeningeal metastases with a single-dose administration of ^186^Re-NL via intraventricular catheter.

### 5.2. “Multicentre Canadian Study to Measure the Safety and Efficacy of Radiosynoviorthesis”

The recently completed multicenter Phase III study (NCT01615991) aimed to measure the safety and efficacy of radiosynoviorthesis performed with ^90^Y-citrate colloid or ^186^Re-sulfide. The ^186^Re-sulfide, manufactured by CURIUM^TM^, is a commercially available product in Europe. The aim of the study was to evaluate the safety of intra-articular administration of both radiopharmaceuticals in patients suffering from arthritis or chronic inflammatory joint disease. A secondary objective was to evaluate efficacy on synovitis (characterized by pain, tenderness, and effusion), which is resistant to systemic therapy and intra-articular corticosteroid injections.

### 5.3. ”Rhenium-Skin Cancer Therapy (SCT) for the Treatment of Non-Melanoma Skin Cancer” (Phase IV, NCT05135052, Recruiting)

One of the non-melanoma skin cancers (NMSC) treatment options is high-dose brachytherapy using a ^188^Re-resin [48,49] commercial name Rhenium-SCT^®^ (Oncobeta^®^ GmbH, Munich, Germany). In particular, in this recent prospective study, the researchers aim to assess the efficacy and safety of a single application of Rhenium-SCT^®^ (Oncobeta^®^ GmbH, Munich, Germany). It is a new therapeutic option that spread the radioactivity on the entire surface of the lesion, making its shape and size non-influencing. Preliminary published results show that this technique is effective in 98% of treated patients [49]. This indicates that this type of non-invasive, easy-to-perform, and tolerable approach appears to be a very viable alternative when surgery or radiotherapy is not possible or rejected by the patient. In the future, larger case series and longer follow-up periods could confirm these preliminary data and help find the optimal personalized dose to reduce early side effects.

### 5.4. “Rhenium-188-HEDP vs. Radium-223-chloride in Patients with Advanced Prostate Cancer Refractory to Hormonal Therapy” (Phase III, NCT03458559, Active, Not Recruiting)

In recent years, a large number of palliative agents for bone pain were developed and are commercially available: [^89^Sr]SrCl_2_ (Metastron^®^), [^223^Ra]RaCl_2_ (Xofigo^®^) [^153^Sm]Sm-EDTMP (Quadramet^®^), and [^186^Re]Re-HEDP(^186^Re-etidronate). The [^186^Re]Re-HEDP formulation had been approved and launched in Switzerland and Greece, but the NDA submitted to the US FDA was never approved and the project in the US was discontinued. Eventually, the product was also withdrawn from the EU countries. In developing countries, generic copies found some interest (mainly because established competitors with IP were too expensive) and the drug is still available outside of the EU and US.

The mechanisms underlying therapies with radiopharmaceuticals containing ^89^Sr and ^223^Ra exploit their natural tropism for bone, as they mimic the activity of the Ca^2+^ cation, while the phosphonates (EDTMP = ethylenediaminetetramethylene phosphonate and HEDP = hydroxyethylidene diphosphonate), labeled with ^153^Sm and ^186^Re, binds to hydroxyapatite crystals by forming hydroxide bridges through the mechanism of chemisorption. Currently, the only one with a proven overall survival benefit is [^223^Ra]RaCl_2_ firstly demonstrated by Biersack et al. in 2011 [50,51,52]. Hence, the launch of the comparative Phase III trial, whose primary endpoint is to compare, in terms of overall survival, the administration of [^188^Re]Re-HEDP or [^223^Ra]Ra-dichloride in patients with castration-resistant prostate cancer metastatic to bone.

### 5.5. “Rhenium ^188^Re-P2045 in Small Cell Lung Cancer and Other Advanced Neuroendocrine Carcinoma” and “^188^Re-P2045 in Patients with Lung Cancer Who Have Received or Refused to Receive Prior Chemotherapy”

^188^Re-P2045 is a somatostatin analogue peptide fragment [53,54] and is the only ^188^Re-labeled peptide-type complex undergoing clinical trials. The aim of the first study (Table 4: NCT02030184, Phase I and II) was to administer the radiopharmaceutical to patients with small-cell lung cancer and other advanced neuroendocrine carcinomas. The study was withdrawn due to renal toxic effects [55,56]. A second trial (Table 4: NCT00100256, Phase I and II), whose status is currently not updated on the “clinicaltrials.gov” portal, involves patients with lung cancer who have received or refused to receive prior chemotherapy [57]. The purpose of this study is to determine the maximum dose that is safely tolerated for the experimental drug ^188^Re-P2045, to learn more about the side effect profile of both ^99m^Tc-P2045 and ^188^Re-P2045 and about the benefit, in terms of lung cancer tumor reduction, as a result of treatment with ^188^Re-P2045. Selected patients will be treated with low ^188^Re-P2045 doses at first to verify if any bad side effects occur. The dose will then be increased when there is confidence that it is safe.

### 5.6. “^188^Re-SSS Lipiodol to Treat HepatoCellular Carcinomas” (NCT0112646, Phase I, Completed)

Hepatocellular carcinoma (HCC) is one of the most common primary cancers in many countries. The strategy that involves the use of ^188^Re-Lipiodol in HCC patients has produced several radiopharmaceuticals [58] over the years, namely ^188^Re-HDD [59], ^188^ReN-DEDC [60], and ^188^Re-SSS [34], but the greater number of clinical trials were conducted with the former [61,62,63,64,65,66,67,68,69,70,71,72,73].

A recent research project coordinated and financed by the IAEA [74] promoted phase I [62] and subsequently phase II [64] trials in several countries. Transarterial radioembolization (TARE) with radiolabeled devices (^90^Y-microspheres: SIR-Sphere and TheraSphere) or radiopharmaceuticals containing Lipiodol (^131^I-microspheres) in HCC intermediate and advanced phases and intrahepatic metastases has demonstrated efficacy and safety [75,76,77]. With reference to this latest radiopharmaceutical, ^188^Re can represent a potentially valid alternative to ^90^Y, thanks to its availability on site and gamma emissions. Replacing intra-arterial hepatic administration of ^131^I-lipiodol with ^188^Re-lipiodol could, beyond clinical outcome, reduce hospitalization to 1 day, and thus reduce costs and improve patient comfort. To date, ^188^Re-HDD is used in several centers in India, but it exhibits low labeling yield and high urinary excretion (over 40% at 72 h) [78,79]. To solve these problems, different compounds, such as ^188^ReN-DEDC-Lipiodol and ^188^Re SSS-Lipiodol, have also been developed, which have shown higher yields and greater stability in vivo [60,80,81]. In France, where HCC mortality on viral cirrhosis C will increase by approximately 150% for men and 200% for women until 2020, this last radiopharmaceutical was developed. It is a ^188^Re^3+^ radiopharmaceutical whose formula is ^188^Re(III)-SSS-lipiodol, where SSS = (S_2_CPh) (S_3_CPh)_2_ [34] (Figure 5). This radiopharmaceuticals type can be prepared quickly both manually and automatically [82,83]. This phase I clinical trial aims to determine the maximum tolerated dose and the recommended ^188^Re-SSS Lipiodol activity for hepatic intra-arterial injection in patients with hepato-cellular carcinoma.

### 5.7. “HER2 Expression Detection and Radionuclide Therapy in Breast Cancer Using ^99m^Tc/^188^Re Labeled Single Domain Antibody (NCT04674722, Phase I, Recruiting)

The overexpression of human epidermal growth factor 2 (HER2) in breast cancer is predictive for an aggressive tumor subtype, a worse prognosis, and a shorter overall survival [84]. Expression of HER2 in a complex environment, such as in a heterogeneous tumor, makes a precise assessment of HER2 status difficult using current methods. For this reason, over the past 10 years, this receptor has been extensively studied as a target for molecular imaging and nuclear medical therapy. In an ideal and modern theranostic approach, the development of radiotracers targeted to HER2 allows a non-invasive evaluation of the expression of HER2 and therefore the selection of patients on which to perform targeted nuclear medical therapies, monitoring their response. In a previous study still, which started in 2019 and is currently in the recruitment phase, (NCT04040686 “HER2 Expression Detection in Breast Cancer Using ^99m^Tc-NM-02”, Phase I,) the safety, dosimetry, and biodistribution of a new single domain antibody were evaluated. ^99m^Tc-NM-02 was synthesized starting from [^99m^Tc(OH_2_)_3_(CO)_3_]^+^ complex that reacted with NM-02 (single-domain antibodies, sdAbs), according to literature [85]. The sdAbs are considered ideal candidates for nuclear medicine applications, because they are small antigens derived from heavy chain antibodies [86]. They are rapidly cleared of the blood, penetrate deep into tissues, and have a great affinity for their antigen [86,87]. This type of ligand was labeled with different radionuclides and proved [85,88,89,90,91,92,93,94,95]. In the phase I study mentioned, which sees ^99m^Tc-MN-02 as the main radiopharmaceutical under evaluation, the preliminary results showed that the tracer accumulated mainly in the kidneys and liver with a slight absorption in the spleen, intestine, and thyroid; while, low levels of background tracer were observed in other organs in which primary tumors and metastases typically occurred [96]. After demonstrating that SPECT ^99m^Tc-NM-02 imaging can be an accurate and non-invasive method for detecting HER2 status in breast cancer patients, a second trial was activated that also provides for the administration of ^188^Re-NM-02. The aim of this second clinical trial, of a distinctly theranostic nature, is to determine the safety and efficacy profiles of the NM-02 antibody fragment labeled with ^99m^Tc and ^188^Re. In particular, the SPECT/CT imaging study of the expression of HER2 will allow to increase the number of cases, while the result of the therapy will be the real goal. The obtained results will be compared with the existing gold standard HER2 expression detection by tissue immunohistochemical biopsy (IHC) and/or fluorescent in-situ hybridization (FISH) method and ^18^F-FDG PET/CT imaging. Breast cancer patients who will be recruited for the study with ^188^Re labeled anti-HER2 sdAb will have positive HER2; progression or relapse after standard treatment, including surgery, chemotherapy, radiotherapy, and targeted therapy; and will receive the radiopharmaceutical in a single dose of injection.

### 5.8. Treatment of Non-Responding to Conventional Therapy Inoperable Liver Cancers by in Situ Introduction of ImDendrim (NCT03255343)

Stereotactic brachytherapy for extensive local tumors offers a very effective treatment option locally without significant complications in medically impaired patients. This latest clinical trial aims to evaluate the efficacy and safety of treatment of non-responding to conventional therapy inoperable liver cancers with ^188^Re-ImDendrim agent, consisting of a poly-L-lysine dendrimer nanovector mixed with complex of ^188^Rhenium-ligand (nitro-imidazole-methyl-1,2,3-triazol-methyl-di-[2-pycolyl] amine). ^188^Re-ImDendrim is composed of a radioactive nitro-imidazole (nitro-imidazolo-metil-1,2,3-triazol-metil-di-[2-pycolil] ammina)-derived ligand loaded with poly-L-lysine dendrimer and is an in-situ delivery system made from dendrimer-diffusible probes for targeting hypoxic tumoral cells combined with the beta emitter ^188^Re-ImDendrim administered by direct stereotaxic intrahepatic injection with guided CT; response to treatment was assessed by standardized quantitative absorption values (SUVs) from [^18^F]-fluorodeoxyglucose (FDG) positron emission tomography/computed tomography (PET/CT). The very preliminary results confirm the safety of ImDendrim and its efficacy. The clinical trial is presumably still ongoing [97]. Recently, the same method was applied in another clinical trial on patients with unresectable lung malignancies [98]. To date, the number of patients subjected to this type of therapy is very small, the first results are positive, but to determine its effectiveness it will be necessary to wait for other studies.

## Figures and Tables

**Table 1:** ^186^Re detailed nuclear decay data [7,9].

	E_β-_ [keV] (Int %)	E_Auger e-_ [keV] (Int %)	E_γ_ [keV] (Int %)	E_X-rays_ [keV] (Int %)
^186^Re→^186^W		6.53 (4.96%)	122.64 (0.603%)	8.4 (1.96%)
		45.7 (0.29%)		57.981 (1.72%)
				59.318 (2.95%)
*Mean energy:*		*1.3 (57%)*	*122.64 (0.603%)*	*46 (8.3%)*
^186^Re→^186^Os	306.1 (21.54%)	6.88(6.55%)	137.157 (9.47%)	8.91(2.92%)
	359.2 (70.99%)	48.3(0.179%)		61.486 (1.14%)
				63.0 (1.94%)
*Mean energy:*	*346.7 (92.59%)*	*1.3 (71%)*	*106 (12.1%)*	*38 (7.3%)*

**Table 2:** ^188^Re detailed nuclear decay data [7,9].

	E_β-_ [keV] (Int %)	E_Auger e-_ [keV] (Int %)	E_γ_ [keV] (Int %)	E_X-rays_ [keV] (Int %)
^188^Re→^188^Os	527.779 (1.85%)	6.88 (6.84%)	155.044 (15.49%)	8.91 (3.04%)
	728.88 (25.8%)	48.3 (0.219%)	478.00 (1.076%)	61.486 (1.40%)
	795.41 (70.7%)		6.33.03 (1.370%)	63.0 (2.38%)
*Mean energy:*	*763 (100.2%)*	*1.3 (75%)*		*40.8 (8.4%)*

**Table 3:** Standard reduction potentials comparison (M = Re, Tc).

Reaction	E°_Re_ [V]	E°_Tc_ [V]	ΔE° [V]
MO_4_^−^ + 3e^−^ + 4H^+^ → MO_2_ + 2H_2_O	0.51	0.74	−0.23
MO_4_^−^ + 7e^−^ + 8H^+^ → M + 4H_2_O	0.36	0.47	−0.11

**Table 4:** Clinical trials involving ^188/186^Re compounds. NA = not applicable [43].

Actual Start/End Study Date and Status	Brief Title	Brief Summary	Study Phase	Condition	Drug/Device	Study Sponsor
January 2000/February 2001 Completed	Biodistribution Study With ^186^Re-labelled Humanised Monoclonal Antibody BIWA 4 in Patients With Adenocarcinoma of the Breast. NCT02204046	To evaluate the safety and tolerability of ^186^Re-bivatuzumab administered intravenously (i.v.) and to study biodistribution and pharmacokinetics in patients with breast adenocarcinoma.	Phase I	Brest Adenocarcinoma	^186^Re-labelled humanized monoclonal antibody BIWA 4	Boehringer Ingelheim
December 1999/February 2001 Completed	Biodistribution Study With ^186^Re-labelled Humanised Monoclonal Antibody BIWA 4 in Patients With Non-small Cell Lung Cancer NCT02204059	To evaluate the safety and tolerability of ^186^Re-bivatuzumab administered intravenously (i.v.) and to study biodistribution and pharmacokinetics in patients with non-small cell lung cancer (NSCLC).	Phase I	Carcinoma, Non-Small-Cell Lung	^186^Re-labelled humanized monoclonal antibody BIWA 4	Boehringer Ingelheim
March 1999/June 2001 Completed	Dose Escalation Study With 99mTC—or 186 Re-labelled Humanised Monoclonal Antibody (hMAb) BIWA 4 in Patients With Head and Neck Cancer. NCT02204033	To evaluate the safety and tolerability of ^186^Re-bivatuzumab administered intravenously (i.v.) and to study biodistribution and pharmacokinetics in patients with Head and Neck Neoplasms.	Phase I	Head and Neck Neoplasms	^186^Re-labelled humanized monoclonal antibody BIWA 4	Boehringer Ingelheim
April 2006/April 2007 Completed	Identification of Sentinel Lymph Nodes With Methylene Blue and Isotope NCT00314405	To evaluate the performance of a double labeling method using isotope and methylene blue dye injection to localize precisely Sentinel Lymph Node (SLN) in a series of 100 patients with infiltrative breast cancer justifying SLN excision.	NA	Infiltrative Breast Cancer	^186^Re-Sulfide (Device)	University Hospital, Strasbourg, France
31 May 2012/31 December 2018 Completed	Multicentre Canadian Study to Measure the Safety and Efficacy of Radiosynoviorthesis NCT01615991	Multicentre Canadian Study to Measure the Safety and Efficacy of Synoviorthesis Performed With ^90^Y- or ^186^Re-Sulfide.	Phase III	Arthritis or chronic inflammatory joint disease.	^186^Re-Sulfide	Centre de recherche du Centre hospitalier universitaire de Sherbrooke
3 June 2015/January 2025 Recruiting	Maximum Tolerated Dose, Safety, and Efficacy of Rhenium Nanoliposomes in Recurrent Glioma (ReSPECT). NCT01906385	Volume and dose escalation study of the safety, tolerability, and distribution of ^186^RNL in patients with recurrent or progressive malignant glioma after standard surgical, radiation, and/or chemotherapy treatment.	Phase I; Phase II	Glioma.	^186^Re-NanoLiposomes (^186^RNL)	Plus Therapeutics
6 December 2021/30 December 2022 Recruiting	Intraventricular Administration of ^186^Re-NanoLiposome for Leptomeningeal Metastases NCT05034497	An open-label Phase I clinical study that will administer a single dose of ^186^RNL via intraventricular catheter for the treatment of Leptomeningeal Metastases (LM).	Phase I	Leptomeningeal Metastasis	^186^Re-NanoLiposomes (^186^RNL)	Plus Therapeutics
26 May 2010/6 August 2019 Completed	^188^Re-SSS Lipiodol to Treat HepatoCellular Carcinomas NCT01126463	To determine the maximum tolerated dose and the recommended ^188^Re-SSS Lipiodol activity for hepatic intra-arterial injection in patients with hepato-cellular carcinoma.	Phase I	Hepatocellular Carcinomas	^188^Re-SSS Lipiodol	Center Eugene Marquis
17 January 2022/15 May 2024 Recruiting	Rhenium-Skin Cancer Therapy (SCT) for the Treatment of Non-Melanoma Skin Cancer. NCT05135052	Efficacy of Personalized Irradiation with ^188^Rhenium-Skin Cancer Therapy (SCT) for the treatment of non-melanoma skin cancer.	Phase IV	Non-melanoma Skin Cancer.	^188^Re-resin (Rhenium-SCT^®^)	OncoBeta International GmbH (OncoBeta Therapeutics)
24 August 2020/30 September 2022 Recruiting	HER2 Expression Detection and Radionuclide Therapy in Breast Cancer Using ^99m^Tc/^188^Re Labeled Single Domain Antibody NCT04674722	To evaluate the safety, dosimetry, and efficacy of ^99m^Tc/^188^Re labeled anti-HER2-single domain antibody (Product Code Name: ^99m^Tc-NM-02 and ^188^Re-NM-02) SPECT/CT imaging of HER2 expression and radionuclide therapy in Breast Cancer.	Phase I	Breast Cancer Radiotoxicity	^99m^Tc or ^188^Re labeled anti-HER2 sdAb.	Shanghai General Hospital, Shanghai Jiao Tong University School of Medicine
16 May 2018/16 May 2024 Active, not recruiting	Rhenium-188-HEDP vs. Radium-223-chloride in Patients With Advanced Prostate Cancer Refractory to Hormonal Therapy. NCT03458559	To investigate if treatment with ^188^Re-HEDP results in an improvement in overall survival compared to treatment with ^223^Ra-chloride.	Phase III	Prostate Cancer Metastatic to Bone.	^188^Re-HEDP vs. ^223^RaChloride	Amsterdam UMC, location VUmc
June 2017/June 2019 Withdrawn	Rhenium 188-P2045 in Small Cell Lung Cancer and Other Advanced Neuroendocrine Carcinomas. NCT02030184	^188^Re-P2045 in small lung cancer and other advanced NE carcinomas.	Phase I; Phase II	Small Cell Lung Cancer (SCLC); Neuroendocrine (NE) Tumors; Large Cell Neuroendocrine (NE) Tumors.	^188^Re-P2045	University of Maryland, Baltimore
January 2004/April 2020 Unknown status	^188^Re-P2045 in Patients With Lung Cancer Who Have Received or Refused to Receive Prior Chemotherapy. NCT00100256	To determine the maximum dose that is safely tolerated for the experimental drug ^188^Re-P2045.	Phase I; Phase II	Lung Neoplasms Carcinoma; Non-Small-Cell Lung Carcinoma; Small Cell Neoplasm Recurrence.	^188^Re-P2045	Andarix Pharmaceuticals
13 March 2017/31 October 2017 Unknown status	Treatment of Non-responding to Conventional Therapy Inoperable Liver Cancers by in Situ Introduction of ImDendrim. NCT03255343	To evaluate the efficacy and safety of treatment of non-responding to conventional therapy inoperable liver cancers by in situ introduction of ImDendrim.	NA	Liver tumor non operable	[^188^Re]Complex Coupled to an Imidazolic Ligand and Associated With a Dendrime (Device)	French Association for the Advancement Medical Research

## Data Availability

Not applicable.
